# Molecular Dynamics Simulation for Evaluating Fracture Entropy of a Polymer Material under Various Combined Stress States

**DOI:** 10.3390/ma14081884

**Published:** 2021-04-10

**Authors:** Naohiro Takase, Jun Koyanagi, Kazuki Mori, Takenobu Sakai

**Affiliations:** 1 Department of Materials Science and Technology, Graduate School of Tokyo University of Science, 6-3-1 Niijuku, Katsushika-ku, Tokyo 125-8585, Japan; 8220521@ed.tus.ac.jp; 2 Department of Materials Science and Technology, University of Science, 6-3-1 Niijuku, Katsushika-ku, Tokyo 125-8585, Japan; 3 Itochu Techno-Solutions Corporation, Art Village Osaki Central Tower, 1-2-2, Osaki, Shinagawa-ku, Tokyo 141-8522, Japan; kazuki.mori.013@ctc-g.co.jp; 4 Department of Mechanical Engineering, Saitama University, Shimo-Okubo, Sakura-ku, Saitama 338-8570, Japan; sakai@mech.saitama-u.ac.jp

**Keywords:** molecular dynamics, entropy, tensile failure, stress triaxiality, void

## Abstract

Herein, the stress-state dependence of fracture entropy for a polyamide 6 material is investigated through molecular dynamics simulations. Although previous research suggests that a constant entropy increase can be universally applied for the definition of material fracture, the dependence of stress triaxiality has not yet been discussed. In this study, entropy values are evaluated by molecular dynamics simulations with varied combined stress states. The calculation is implemented using the 570,000 all-atom model. Similar entropy values are obtained independently of stress triaxiality. This study also reveals the relationship between material damage, which is correlated with void size, and the entropy value.

## 1. Introduction

Carbon fiber reinforced polymers (CFRPs) are used not only in the aerospace field but also in an increasing number of products such as automobiles [1]. However, prediction of the lifetime of CFRPs is difficult, thereby limiting their application [2]. This difficulty arises because the damage to CFRPs is more complex than that to metallic materials as it is caused by microscopic damage, such as carbon fiber breakage [1,3], microcracks in the resin [4,5], and delamination of the interface between the carbon fiber and resin [6,7]. Thus, it is difficult to quantitatively measure the lifetime of CFRP-based materials. In addition, the time dependence of damage is known to exist in polymeric materials [8,9,10]. Therefore, mechanisms of the damage occurring at the molecular level must be considered for the lifetime prediction of materials. In this study, we propose that the lifetime of materials can be measured by entropy. The entropy is determined by the dissipated energy over the absolute temperature, and the dissipated energy is determined by the stress–strain history [11]. Thus, the entropy is a mechanical value obtained from a mechanophysical quantity. Previous studies have utilized the increase in entropy of metallic materials as a criterion of lifetime prediction for nickel [12,13], aluminum [14,15,16], and steel [17,18]; however, no such study has been conducted on polymeric materials. If entropy can be used as a lifetime criterion, we propose that it can be applied to multiscale analysis, combining molecular dynamics and finite element methods in the future. By incorporating both material parameters and lifetime entropy obtained by molecular dynamics into the finite element method, we believe that structural analysis can be performed by considering the molecular structure of the material.

Furthermore, external loads cause damage to the materials, which increases the entropy inside the material. When a constant entropy increase is reached, the material fails [11]. The entropy at which the material fails is defined as the “fracture entropy”; thus, it is possible to use the fracture entropy to predict the lifetime of a material under various loads. According to these theories, the material is expected to fail with fracture entropy under any load. In addition, the resin near the fiber interface in CFRPs is subjected to a combined stress state [6]. Thus, it is important to confirm that the resin will fail at a constant entropy increase under stress from any direction.

Therefore, the purpose of this study is to compare the entropy increase at failure with different combined stress states and to investigate whether or not the material fails at a constant entropy increase in all simulations. In addition to reproducing the damage mechanism at the molecular level, molecular simulations allow us to consider thermodynamic parameters, such as internal energy [19,20], which are difficult to obtain experimentally, in addition to temperature [21,22], interface energy [23,24], and mechanical properties [25,26,27]. At the same time, we propose a method for calculating entropy, which has recently been used in a discussion of molecular dynamics simulations [28,29,30,31,32,33]. In particular, the entropy calculation method proposed in this study can also be applied to mechanical experiments of materials.

## 2. Method

### 2.1. Polymer System

**Figure 1:**
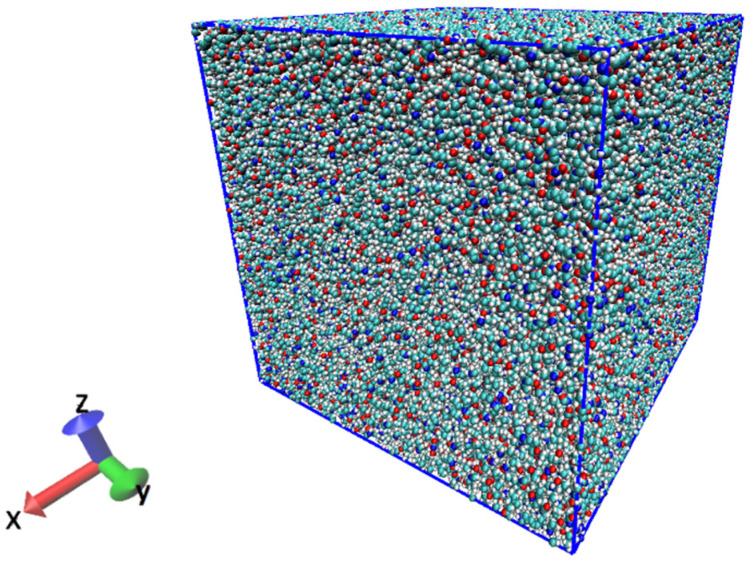
Illustration of the initial system of PA6 consisting of 1000 molecules.

In this study, fracture simulations were performed on a single polyamide 6 (PA6) resin under different conditions. The all-atom model of PA6 was used, the degree of polymerization was set to 30, and the number of atoms was 570,000. After randomly arranging the molecular chains in the cell, the system was set to 650 K, which is above the melting point of PA6. Next, annealing was performed at 1 atm with isothermal–isobaric ensemble (NPT). In annealing, the volume of the resin decreases. The NPT ensemble was used to reduce the volume of the system and to keep the pressure at 1 atm to make the system stable. The system was annealed at a cooling rate of 70 K/ns to an ambient temperature of 300 K. We conducted the relaxation in NPT ensemble (1 atm, 300 K) for 10 ns and ensured that the system density was 1.1 g/cm^3^ and the total energy was stable. The Nose–Hoover method [34] was used for temperature control. The system used periodic boundaries to avoid wall effects. The system created from the above process is shown in Figure 1. The cell size is a cube of 17.4 nm per side.

### 2.2. Fracture Simulation

Fracture simulations were performed using the microcanonical (NVE) and isoenthalpic-isobaric (NPH) ensembles. To calculate the entropy, it is necessary to obtain the temperature increase of the system due to pulling. Entropy is one of the energy-related values. In order to make this discussion simple, the energy flow between inside and outside simulation cell has to be zero during fracture simulation. Therefore, we used the NPH and NVE ensembles. The ensemble was changed only to manipulate the stress triaxiality in fracture simulation; there is no difference between the NPH and NVE ensembles except for the stress state in the fracture phenomena. In the NPH ensemble, the pressure in the *x* and *y* directions was maintained by the Parrinello–Rahman method [35]. In the NVE ensemble, the cell size in the *x* and *y* directions was fixed for triaxial pulling. Four different tensile simulations with different stress triaxialities were performed to determine the difference in damage. The stress triaxiality is a parameter that represents the state of loading. For example, *η* = 0 indicates shear tension, and *η* = 1/3 indicates uniaxial tension. The equation for stress triaxiality is shown in Equation (1).

(1) $$\eta =\frac{\sigma_{m}}{\bar{\sigma }}$$

where

(2) $$\sigma_{m}=\sqrt{\frac{{\left(\sigma_{1}-\sigma_{2}\right)}^{2}+{\left(\sigma_{2}-\sigma_{3}\right)}^{2}+{\left(\sigma_{3}-\sigma_{1}\right)}^{2}}{2}}$$

and

(3) $$\bar{\sigma }=\frac{\sigma_{x}+\sigma_{y}+\sigma_{z}}{3}$$

where *η* is the stress triaxiality; $\sigma_{m}$ is the von Mises stress; $\bar{\sigma }$ is the hydrostatic stress; $\sigma_{1}$, $\sigma_{2}$, and $\sigma_{3}$ are the principal stresses; and $\sigma_{x}$, $\sigma_{y}$, and $\sigma_{z}$ are the axial stresses.

The conditions for each experiment are listed in Table 1. Compression and tension are produced by deforming the cell. Owing to the constraints of the simulation, the tension in the z-direction was set to a constant strain rate of 10^8^/s (engineering strain). However, the strain rate in the simulation was much higher than the experimental strain rate. The pressure in the XY direction is set to achieve the optimum stress triaxiality by repeating the simulation with different values of pressure. GROMACS 2018.3 [36] was used for the molecular dynamics simulations. The force field was all-atom optimized potentials for liquid simulations (OPLS-AA) [37], and the parameters were created using PolyPerGen [38]. The functional functions and basis functions for structure optimization were performed using B3LYP/6-31 G*. Based on the optimized structure, the functional functions and basis functions of the electric charge calculation were performed utilizing MP2/ 6-31 G*. The long-range interactions were calculated using the particle mesh Ewald method [39] with a grid size of 0.12 nm. The intermolecular interaction cutoff was set to 1.0 nm. The initial temperature of the system was set to 300 K. The LINCS algorithm [40] was used to implement the MD simulations for time increments of 2 fs. The calculations were implemented using a cloud-based computer provided by Exabyte.io and GPU P100 supplied by Azure [41].

### 2.3. Fracture Entropy Calculation Method

Two methods were used to calculate entropy in the molecular simulation: thermodynamic and mechanical. The entropy calculation equation by the thermodynamic method is shown in Equation (4), which is based on the second law of thermodynamics. The external force work is calculated from the stress and displacement in each XYZ plane. In addition, temperature T is the temperature of the system at each time.

(4) $$S=\int_{T_{0}}^{T_{1}}\frac{dQ}{T}dT$$

where

(5) dQ=dU−PdV

where *S* is the entropy, *Q* is the heat, *T*_1_ is the temperature at fracture, *T*_0_ is the initial temperature, *P* is the pressure, *V* is the volume, and *U* is the internal energy. The internal energy in Equation (4) is obtained by summing the kinetic energy and potential energy of the atom. The potential energy is the sum of the Coulomb potential, the Lennard-Jones potential, and the intramolecular potential defined by OPLS-AA [37].

The equation for calculating entropy using the mechanical method is shown in Equation (6) [11]. The inelastic strain energy was obtained by dividing the elastic strain energy by the total strain energy. It was also calculated for each of the XYZ planes, and the total value was used as the inelastic strain energy of the entire system. It should be noted that this equation can be used not only for molecular simulations but also for experiments.

(6) $$\gamma_{f}=\int_{0}^{t_{f}}\left(\frac{W_{p}}{T}\right)dt$$

where

(7) $$W_{p}=W\;-\;W_{e}$$

and

(8) $$W=\int_{0}^{\varepsilon }\sigma d\varepsilon$$

where *γ_f_* is the fracture entropy, *t* is the elapsed time from the start of pulling, *t_f_* is the time to fracture defined as the time from the commencement of tension until yielding and the value of stress reaches zero, *T* is the temperature, *W* is the strain energy, *W_p_* is the inelastic strain energy, *W_e_* is the elastic strain energy, and *ε* is the strain. The elastic strain energy should be considered for the value of entropy before the failure. However, the elastic strain energy has no effect on the fracture entropy because the stress reaches zero when failure occurs. In this study, the elastic strain energy is ignored.

### 2.4. Measurement of Void

To detect the voids, our own software was used in combination with Visual Molecular Dynamics (VMD) 1.9.3 [42]. First, VMD was used to obtain the lattice data representing the vacant spaces in the system. The lattice spacing was set to 0.1 nm, and a vacant space was defined as the absence of atoms within a radius of 0.3 nm from the lattice point. The lattice points of neighboring vacant spaces were grouped using our software, and the groups were defined as voids. The center of the voids and volume of each void were calculated and displayed on the VMD.

## 3. Results and Discussion

### 3.1. Progress of Fracture

**Figure 2:**
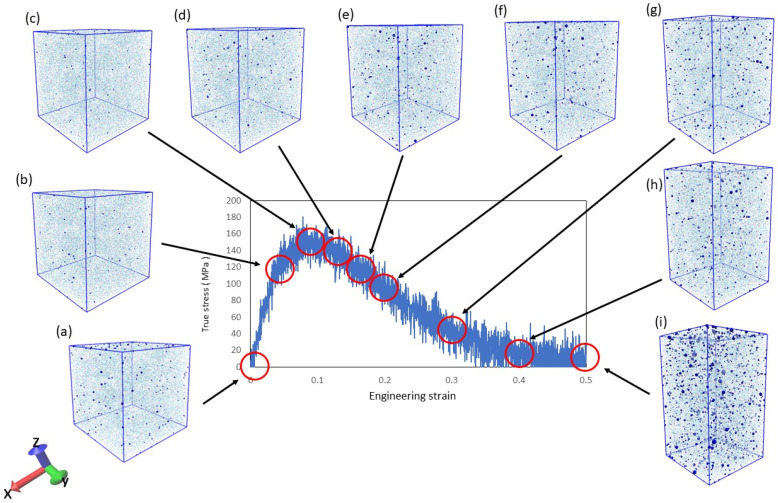
Stress–strain relationship with void increase due to fracture progression. (a–i) corresponds to the illustration of the voids in Figure 3.

**Figure 3:**
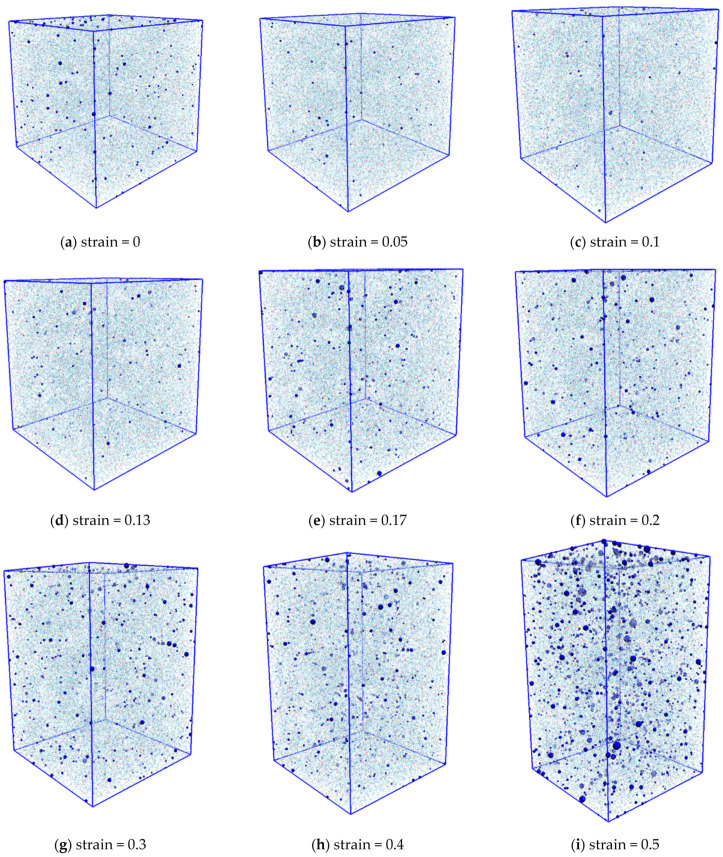
The generation and growth of voids inside the resin at each strain.

In the tensile simulations, we considered whether damage occurred to the resin. It is known that resin damage corresponds to the generation and growth of voids on the microscale [43]. Therefore, it can be seen in Figure 2 and Figure 3 that increasing the strain of the resin in the simulation increases the number of voids. In Figure 2, (a–i) corresponds to the illustration of the voids in Figure 3. The system used to verify the voids is the uniaxial tension condition shown in Table 1.

**Figure 4:**
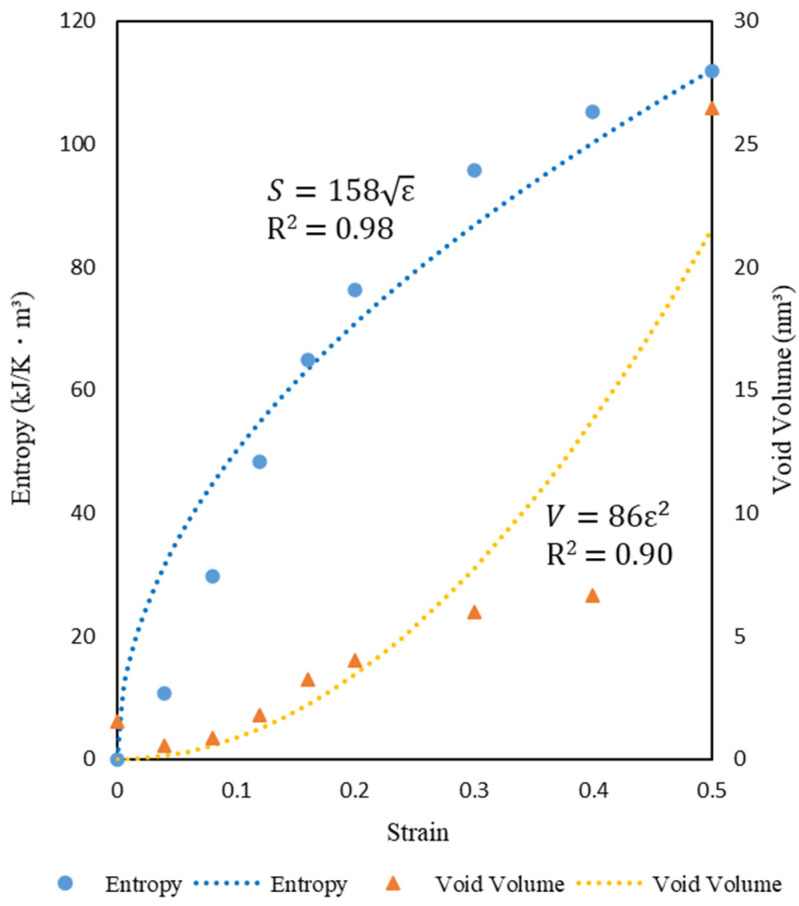
Relationship of entropy and void volume with strain.

The blue dots indicate voids, and the number and size of voids increase with increasing strain. This indicates that damage was generated by the simulation. Furthermore, entropy increases with increasing void volume, as shown in Figure 4.

The results show entropy increasing with the increase in voids. However, an exception is seen when the strain increases from 0.0 to 0.04; the void volume decreases with respect to the entropy increase. Additionally, at strain 0.5, the entropy increase is slower than the rapid increase in the void volume. These results will be discussed and compared with experimental results in the future.

Next, we conduct a regression analysis and suggest the following equation to explain entropy and void volume in Equations (9) and (10).

(9) $$S=a\sqrt{\varepsilon }$$

(10) $$V=b\varepsilon^{2}$$

where *S* is entropy, *ε* is strain, R^2^ is the coefficient of determination, *V* is void volume, and *a*, *b* are empirical coefficients. The coefficient of determination is closer to 1, which means that the regression curve is more suitable for the data. Both R^2^ values are close to 1 and that these equations are suitable to explain entropy and void volume. Furthermore, we suggest the equation relating entropy to void volume from Equations (9) and (10) in Equation (11).

(11) $$S=\alpha \sqrt[{4}]{V}$$

where *α* is empirical coefficient. This equation determines the entropy from the void volume for a material whose loading history is unknown. It can be used to measure the lifetime of materials whose loading history is unknown. However, it is unclear whether this equation is suitable for explaining entropy and void volume. Further discussion of this equation is needed.

### 3.2. Difference of Stress Triaxiality

**Figure 5:**
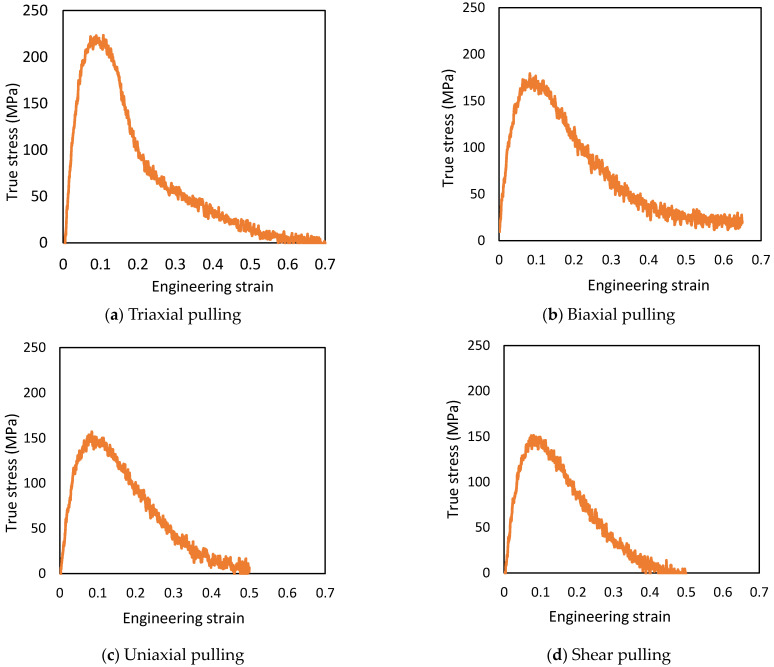
Stress–strain curves with different combined stress states (z-axis).

The true stress is defined as $\sigma_{z}=\;-P_{zz}$ from the pressure tensor, with the simulation running until the stress value reaches 0 MPa after yielding. The strength and stress triaxiality results are listed in Table 2, and the stress–strain curves are shown in Figure 5. The strength of triaxial pulling was 220 MPa at *ε* = 0.11, biaxial pulling was 176 MPa at *ε* = 0.10, uniaxial pulling was 156 MPa at *ε* = 0.08, and shear pulling was 149 MPa at *ε* = 0.09. The strain at break was *ε* = 0.70 for triaxial pulling and *ε* = 0.50 for uniaxial pulling and *ε* = 0.50 for shear pulling, where *ε* is strain. In the case of biaxial pulling, the simulation error prevented pulling until the time of fracture, but the strain at break was estimated to have fractured at a strain of *ε* = 0.65, at which the change in stress was almost constant.

### 3.3. Fracture Entropy Calculation

**Figure 6:**
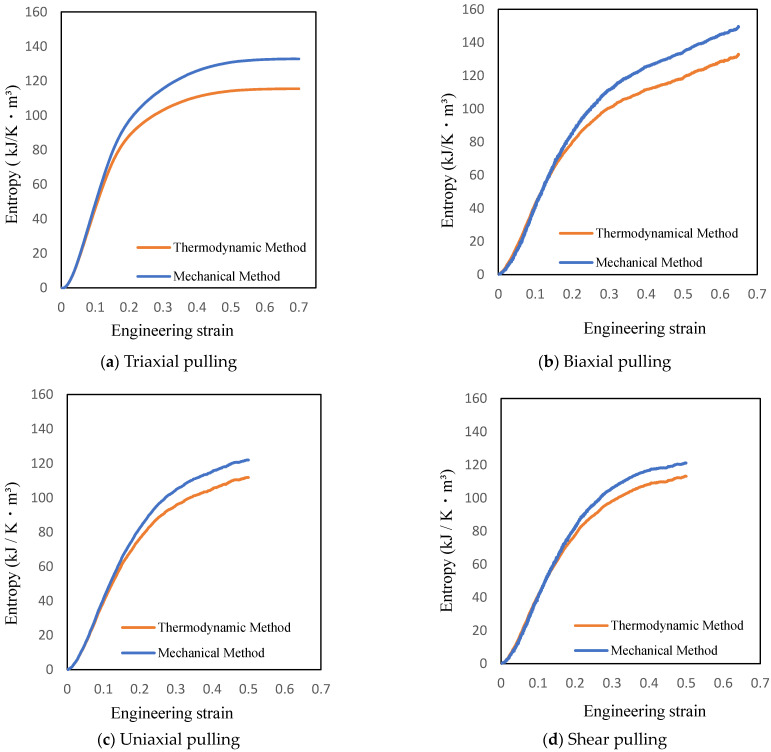
Entropy increase with different combined stress states using two different methods.

The entropy equations by the thermodynamic and mechanical methods are shown in Figure 6, exhibiting the increase in fracture entropy.

Equation (4) from the thermodynamic method and Equation (6) from the mechanical method both can be utilized for determining entropy. Therefore, it is assumed that the entropy values given by the two equations are equal. However, the results in Figure 6 show that the entropy increases differently for the two methods. This difference is attributed to the accumulation of large errors in the stress values determined by the virial equation [23] in mechanical method. Thus, it is possible to calculate the increase in entropy, a thermodynamic physical quantity, using the stress and strain values determined by the mechanical method, allowing entropy damage of the actual structure to be tracked.

The initial resin before tension is expected to have a homogeneous density. The entropy is expected to be low because the polymer chains are entangled and stuck together. If the resin is pulled, the entire density of the resin is expected to decrease, and voids are generated. In this process, the degree of freedom of the molecules increases due to the untangling of the polymers. We believe that this increase in the degree of freedom causes an increase in entropy. In addition, voids are regarded as damage. From the above, we believe that an increase in entropy causes an increase in voids.

Based on the thermodynamic method shown in Equation (4), entropy is a physical quantity corresponding to specific heat. Additionally, damage can be caused by tensile or compressive stress; however, on the micro scale, it is thought to be caused by the movement of molecules and the untangling of molecular chains. This increase in the degree of freedom of the molecular chain, in turn, increases its heat capacity. Thus, it can be said that damage is an increase in the degree of freedom of the molecular chain, which can be regarded as an increase in entropy, a physical quantity corresponding to heat capacity.

## 4. Conclusions

The stress-state dependence of fracture entropy for a polyamide 6 material is investigated through molecular dynamics simulations. To achieve this, entropy values are evaluated by molecular dynamics simulations with varied combined stress states. Similar entropy values are obtained independently of stress triaxiality. This study also reveals the relationship between material damage, which is correlated with void size, and the entropy value. We believe the increase in the degree of freedom in the molecular chain caused by material damage increases the entropy value.

## Figures and Tables

**Table 1:** Simulation conditions for fracture simulation.

Stress State	Ensemble	Stress Triaxiality	Setting Pressure	Setting Pressure	Strain at Fracture
			(X-axis) (MPa)	(Y-axis) (MPa)	
Triaxial pulling	NVE	1.81	-	-	0.70
Biaxial pulling	NPH	0.68	−200	−200	0.65
Uniaxial pulling	NPH	0.26	1	1	0.50
Shear pulling	NPH	0.08	−100	−100	0.50

**Table 2:** Simulation results: relationship between stress triaxiality, maximum stresses, strain at fracture, and fracture entropy.

Stress State	Ensemble	Stress Triaxiality	Maximum Stress (Z-axis) (MPa)	Strain at Fracture	Fracture Entropy
					(Thermodynamical Methods) (kJ/Km³)
Triaxial pulling	NVE	1.81	220	0.70	115
Biaxial pulling	NPH	0.68	176	0.65	131
Uniaxial pulling	NPH	0.26	156	0.50	111
Shear pulling	NPH	0.08	149	0.50	114

## Data Availability

This study did not report any data.
